# Climate change and cancer care: impacts and implications for patients and healthcare professionals

**DOI:** 10.3332/ecancer.2025.1973

**Published:** 2025-08-22

**Authors:** Calvin R Flynn, Roselle de Guzman, Olubukola Ayodele, Joan H Schiller, Katie Lichter, E Shelley Hwang, Lisa Fox, Gustavo Gosling, Claire Hopkins, Ken Rogan, Eduardo Cazap, Seamus O’Reilly

**Affiliations:** 1 Cork University Hospital, Wilton, Cork, Ireland; 2 Manila Central University – FDTMF Hospital, Metro Manila, Caloocan City 1400, Philippines; 3 University Hospitals of Leicester NHS Trust, Leicester, UK; 4 Oncology Advocates United for Climate and Health – International, Madison, WI, USA; 5 Department of Radiation Oncology and Applied Sciences, Dartmouth Hitchcock Medical Center, New Hampshire, USA; 6 Duke University Medical Center, Durham, NC USA; 7 The Institute of Cancer Research, ICR CTSU, 15 Cotswold Rd, London, UK; 8 Latin American Cooperative Oncology Group, Porto Alegre, Brazil; 9 Cancer Trials Ireland, Royal College of Surgeons in Ireland, 123 St Stephens Green, Dublin, Ireland; 10 Latin-American and Caribbean Society of Medical Oncology, Buenos Aires, Argentina; a http://orcid.org/0000-0001-5266-777X; b http://orcid.org/0000-0002-1930-9935; c http://orcid.org/0000-0003-3809-528X; d http://orcid.org/0000-0003-4136-6490; e http://orcid.org/0000-0002-8571-1148; f http://orcid.org/0009-0008-9749-3185; g http://orcid.org/0000-0001-8632-6826; h http://orcid.org/0000-0002-2887-7336

**Keywords:** climate change, cancer care, oncology, sustainability, global health

## Abstract

Caring for patients with cancer is now being conducted in the era of a triple planetary crisis, which threatens our future on the planet. The impacts of this crisis are profound – disrupting cancer care due to displacement from extreme weather events, increasing cancer incidence and worsening cancer outcomes due to pollution, and threatening food and economic security due to loss of biodiversity. Clear that these changes will worsen in the coming years. The burden of these changes are not equitable, with the greatest impacts on countries that have contributed least to the crisis. While healthcare is the fifth leading cause of greenhouse gas emissions, climate awareness and activism in healthcare are low.

This paper examines the relationship between climate change and cancer care, highlighting regional disparities, environmental drivers of cancer risk, and the need for systemic adaptation. We present case studies from the Philippines, Nigeria, and Brazil each illustrating how climate-related events affect oncology delivery in vulnerable settings. We then explore how pollution, heat, and plastic use intersect with cancer risk and outcomes. Finally, we outline practical strategies and evidence-based toolkits for decarbonising cancer care across surgery, radiotherapy, medical oncology, and clinical trials. These insights, informed in part by global collaboration during London Global Cancer Week 2024, support the urgent integration of sustainability into oncology practice worldwide.

## Background

Humanity now faces three interlinked threats: climate change, pollution and biodiversity loss [1]. Overuse of the planet’s existing resources now means that we are taking from (colonising) the resources needed by our families in the future [2]. Consequently, we now live and practice medicine in the era of “temporal colonialism”. This era was first recognised by the scientist Rachel Carson in her landmark publication ‘*Silent Spring*’, a book whose title was derived from the absence of birdsong, which normally heralds spring but was absent due to pesticide-related avian deaths [3]. In her book, she presciently wrote ‘We stand now where two roads diverge, but unlike the roads in Robert Frost’s familiar poem, they are not equally fair. The road we have long been travelling is deceptively easy, a smooth superhighway on which we progress with great speed, but at its end lies disaster’. A million copies of the book were sold before Rachel died of metastatic breast cancer in 1964.

Rachel Carson was correct. In the decades since her death, economic losses from climate change are estimated at $23 trillion due to premature mortality, healthcare expenditure and healthcare-related work loss [1]. Over 570,000 deaths have resulted from climate change-intensified weather events in the past two decades [4], while between 2016 and 2021, climate change-related events displaced over 134.1 million people, including 43.1 million children [5]. This is our new reality. The amount of carbon dioxide that would need to be removed by 2100 to limit global warming to its current 1.5^o^C level is equivalent to running the United States (US) energy industry in reverse for around 80 years [6].

While this Anthropocene era has seen significant gains in life expectancy, these advances have paradoxically come at great environmental cost to the environment. Modern healthcare produces the same global emissions as Africa, a continent of 1.5 billion people in 54 countries [7]. If we are to move beyond a ‘lifeboat ethic’ [8] to one of effectively reducing climate change, marked global behaviour change will be required [9], including changes in the professional and personal behaviour of our healthcare community [10–13]. A multinational survey of 4,654 healthcare professionals assessing views on climate change as a human health issue demonstrated high awareness of the health implications of climate change, but barriers existed to engagement in advocacy and education [14]. These findings, combined with the growing clinical relevance of climate change and its documented impact on cancer outcomes [15–29], underscore the urgency of climate adaptation within oncology.

This paper aims to explore the complex relationship between climate change and cancer care, highlighting the effects on healthcare systems, patient outcomes, and clinical practice. We begin with case studies from the Philippines, Nigeria, and Brazil to illustrate how climate change manifests across diverse geographic and socioeconomic settings. We then examine broader environmental threats—particularly air pollution and plastic use—before outlining evidence-based strategies for reducing the carbon footprint of cancer treatment across surgical, radiation and medical oncology. Finally, we present practical toolkits and policy frameworks to support healthcare professionals and institutions in transitioning toward more sustainable oncology practices. Insights shared during a 2024 international webinar convened by Cancer Trials Ireland, the Latin-American and Caribbean Society of Medical Oncology, Latin America Cooperative Oncology Group, and Oncologists United for Climate and Health International helped inform the structure and priorities of this paper, offering frontline perspectives and reinforcing the need for global collaboration on climate-adaptive cancer care.

## Regional impacts of climate change on communities

### The Philippines

The Philippines is one of the most disaster-prone countries in the world. Located on the Pacific Ring of Fire, the country is highly susceptible to seismic and volcanic risks. Its 7,000 islands sit in the middle of the world's most storm-prone region, the Northwestern Pacific Basin, which is the most active tropical cyclone basin. The Philippines experiences an average of 20 cyclones per year, with some of the most severe typhoons making landfall. Climate change is exacerbating these risks.

The country has one of the world’s longest coastlines, and rapid sea-level rise is occurring at three times the global average [30]. This adds to the vulnerability of the coastal communities that lie directly in the path of storms. Projections reveal that sea levels in the Philippines will continue to rise at a rate slightly above the global average, with an estimated increase of approximately 20 cm by the end of the 21st century [31]. Millions of Filipinos living in coastal communities are at risk, and an estimated 13.6 million people may require relocation [30]. In 2024 alone, the Philippines recorded 2.5 million internal displacements due to natural disasters [32].

The heat index in several regions of the Philippines rose above 42°C (108°F) during the dry months of 2024 as a result of global warming [33]. The highest heat index ever recorded was 55°C in Guiuan, Eastern Samar, in May 2024 [33]. The average temperature across the Philippines is projected to increase by 0.9°C–2.3°C by the mid-21st century [31].

**Figure 1.**
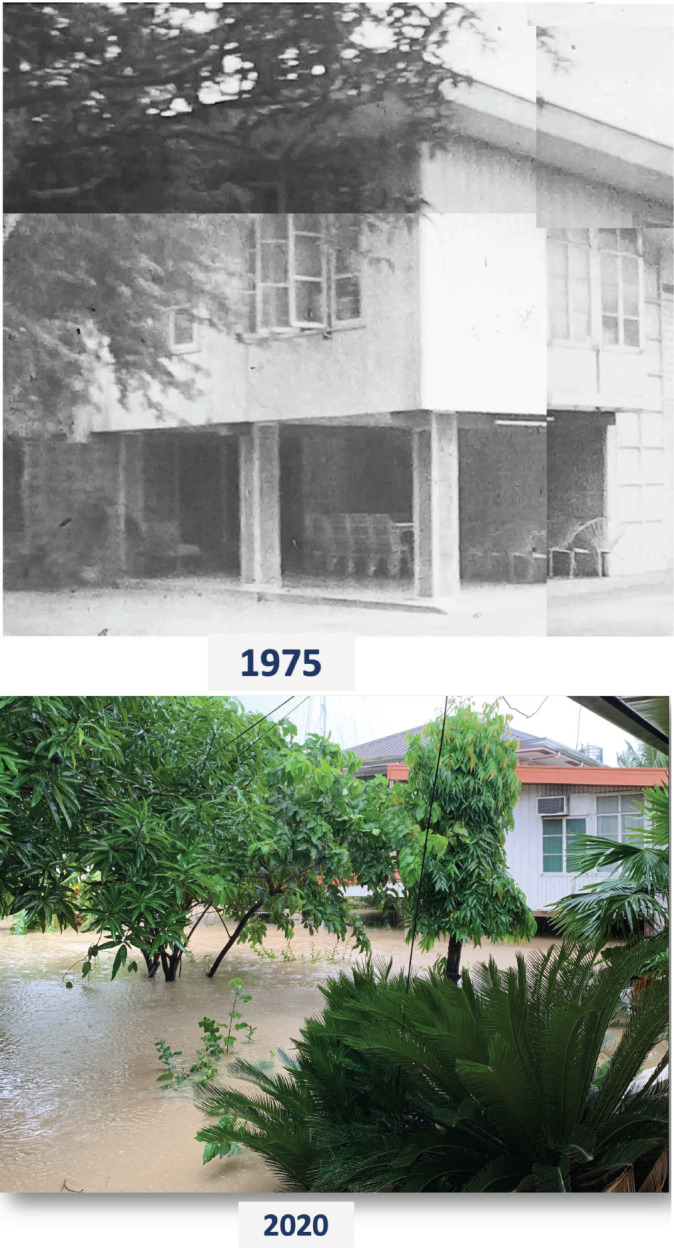
Images of family home in Philippines in 1975 and subsequently in 2020.

The Philippines consistently ranks among the countries with the highest disaster risk, holding the top spot in 2024 [34]. This reflects the country’s exposure to natural hazards and its limited capacity to adapt and respond. Contributing factors include insufficient infrastructure and inadequate coping mechanisms within vulnerable communities. Healthcare infrastructure is particularly at risk from extreme weather events. It is projected that around 550 of the 2,057 hospitals in the Philippines (26.7%) could face partial or total shutdown by 2100 [35]. Many hospitals are unlikely to withstand severe weather events, such as riverine flooding, coastal inundation, and surface water flooding (Figure 1).

### Nigeria

While the Philippines faces the brunt of tropical cyclones and rising sea levels, Nigeria grapples with urbanisation-driven flooding and air pollution. Although the environmental challenges differ, both countries reflect how climate change disproportionately affects low- and middle-income regions, exacerbating health inequities and straining healthcare infrastructure.

Climate change poses significant challenges for low-income countries like Nigeria, manifesting through flooding, extreme weather and pervasive air pollution. These environmental shifts threaten water quality, food security and public health. The intensification of human activities, notably industrialisation and urbanisation, has markedly increased in Nigeria, thereby exacerbating already severe weather conditions. Current projections indicate that by 2030, over 60% of Nigeria’s populace is anticipated to reside in urban areas, a demographic shift that is likely to further elevate temperatures and amplify the frequency of heatwaves, air pollution and flooding incidents [36].

**Figure 2.**
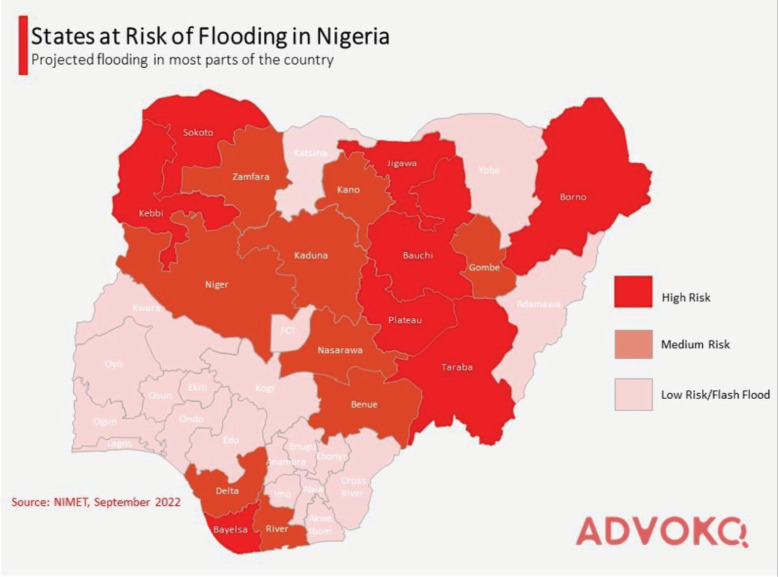
Flooding vulnerability in Nigeria.

The issue of flooding has been particularly acute in Nigeria, displacing more than 500,000 individuals. The Nigerian Meteorological Agency highlighted in 2022 that regions adjacent to the River Niger and Benue face heightened risks of flooding [37] (Figure 2). Additionally, the northern regions of the country have reported more than a 50% increase in rainfall, positioning them precariously for impending disasters. This has been starkly illustrated by the flooding events in Borno State in October 2024, which resulted in 300 fatalities and impacted approximately 1.2 million people [38].

Moreover, the dual processes of urbanisation and industrialisation have precipitated significant air quality degradation in Nigeria, evidenced by elevated levels of particulate matter and other carcinogenic pollutants. Notably, the Niger Delta region, particularly in Port Harcourt, has reported alarming levels of black soot, primarily linked to crude oil extraction and refining activities. Several studies indicate that exposure to this pollution has been associated with deleterious health outcomes, including respiratory ailments, skin disorders and reproductive health issues, affecting over 20,000 individuals. The most concerning respiratory issues include an increase in cases of *EGFR*-mutated lung cancer [39, 40].

The intersection of flooding and agricultural practices further complicates the landscape, as flooding events can lead to the contamination of water sources. This concern is heightened by the escalating use of pesticides and agrochemicals, which are detrimental to infrastructure, arable land and livestock. The resultant contamination exposes communities to carcinogenic substances through both water and food sources, increasing the risk of gastrointestinal malignancies [41–43].

**Figure 3.**
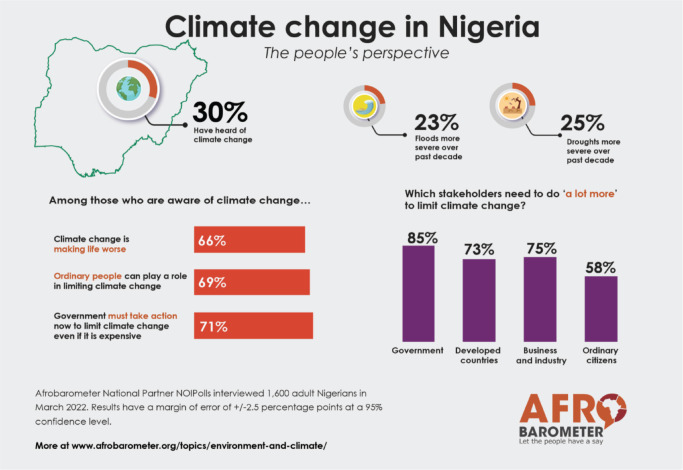
Public perspectives of climate change in Nigeria.

A survey conducted by Afrobarometer in March 2022, involving 1,600 adult Nigerians, revealed that two-thirds (66%) of the respondents are aware of the deteriorating effects of climate change on the nation. However, a dominant majority (85%) believes that the onus of addressing climate change predominantly falls upon governmental entities and developed countries [44] (Figure 3). While it is imperative for national governments to implement stringent environmental regulations, advocate for sustainable energy solutions and curtail greenhouse gas emissions, it is equally vital to promote community awareness regarding the nexus between climate change and cancer. Community-led initiatives for environmental stewardship are essential for fostering impactful responses to these multifaceted challenges.

### Brazil

**Figure 4.**
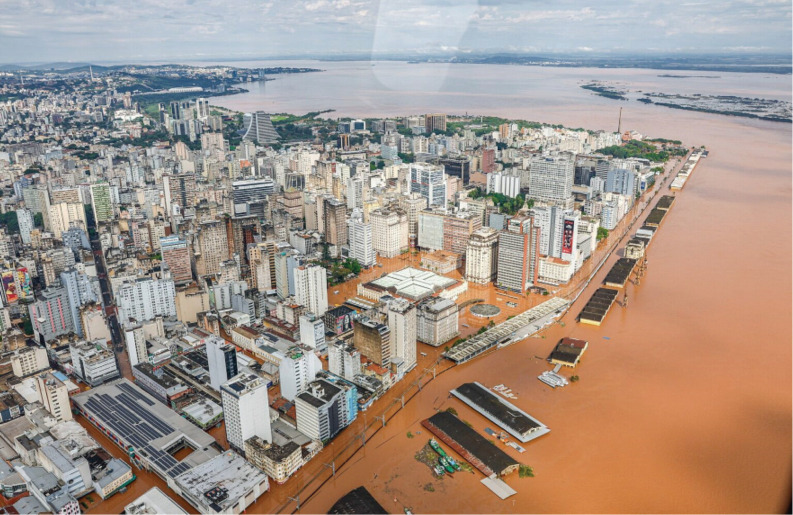
Aerial view of flooding in Porto Allegre May 2024.

**Figure 5.**
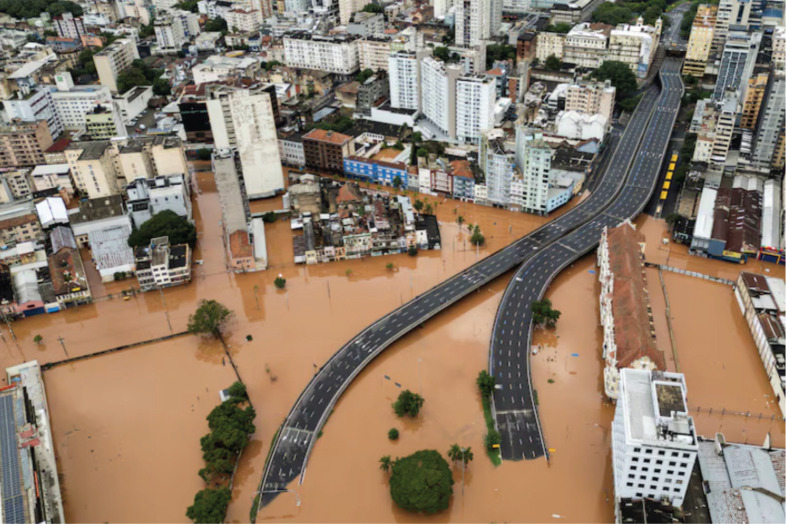
Impact of flooding on critical infrastructure preventing access to care.

Like Nigeria, Brazil faces the dual crisis of climate change and environmental degradation, with extreme weather events increasingly threatening healthcare infrastructure and cancer care delivery. The catastrophic floods that swept through southern Brazil in May 2024 have left an unprecedented humanitarian and healthcare crisis in their wake. Rio Grande do Sul, a state larger than the UK, was hit hardest, with entire communities submerged, critical infrastructure destroyed and over 580,000 people displaced [45] (Figures 4 and 5). Hospitals were forced to evacuate critically ill patients by helicopter or boat, while many were left without access to chemotherapy, dialysis or life-saving medications due to the widespread collapse of transportation and supply chains [46]. The impact on cancer care was particularly severe, with oncology units closing and treatment delays putting thousands of vulnerable patients at risk [47].

Beyond immediate disruptions, the floods have compounded pre-existing health inequities. The devastation disproportionately affected marginalised communities, particularly Black and working-class populations living in high-risk urban peripheries [48]. These communities, already burdened by systemic health disparities, now face increased risks of waterborne diseases like leptospirosis and dengue, as well as food insecurity and long-term mental health consequences [46]. The loss of medical records, supply shortages and power outages have further compromised continuity of care, placing cancer patients at heightened risk. Addressing the psychological toll of climate disasters is critical, as recent research highlights the importance of trauma-informed care and mental health interventions for affected populations [49].

Despite years of warnings from climate scientists, insufficient infrastructure investments, environmental policy rollbacks and weak urban planning left Brazil unprepared for such an event. The Lancet Countdown Latin America report highlights gaps in climate adaptation policies at the city level [50], which may have contributed to challenges in coordinating emergency response efforts in Porto Alegre and other affected areas.

To build resilience in cancer care, Brazil must urgently integrate climate adaptation into health policy. Disaster preparedness plans tailored to oncology, strengthened supply chains and regional coordination strategies are essential to maintaining care continuity during extreme weather events. The National Comprehensive Cancer Network Framework for Resource-Stratified Guidelines provides a model for prioritising oncology services in resource-constrained settings, helping to safeguard cancer patients amid climate disasters [51]. Implementing such frameworks within national disaster response plans will be critical in protecting vulnerable populations from the accelerating threats posed by climate change.

### Interaction of climate change, air pollution and cancer

Beyond localised flooding and extreme weather events, climate change drives widespread environmental degradation, with air pollution emerging as a leading global health threat. This intersection of climate change, pollution and public health has profound implications for cancer incidence and outcomes, affecting vulnerable populations worldwide.

In 2021, the World Health Organisation called climate change ‘the single biggest health threat facing humanity’ [52]. A joint editorial across 230 publications, including the *New England Journal of Medicine* and *The Lancet* stated that the health effects of climate change are ‘catastrophic’ [53], while the UN Secretary-General Antonio Guterres warned that humanity has ‘opened the gates of hell’ [54]. The Lancet Commission on Public Health emphasised that ‘Pollution... is an existential threat to human health and planetary health’ [55]. However, climate change is often perceived by the public as an environmental, political or social issue. Overlooked is the significant impact of climate change and air pollution on healthcare delivery, outcomes and cancer care specifically.

Air pollution and climate change are two sides of the same coin: they are both driven by the burning of fossil fuels. These fossil fuels emit so-called greenhouse gases (CO_2_, nitrogen dioxide, methane and hydrofluorocarbons, among others) that accumulate in the troposphere, trapping solar radiation and raising global temperatures. Over the past 150 years, greenhouse gas emissions have risen exponentially, correlating with increasing global temperatures. This warming trend is associated with extreme heat, severe weather events, shifts in vector ecology (fleas, mosquitoes and ticks), rising sea levels and water and food shortages [56, 57]. These changes disproportionately affect poorer, underserved communities that contribute the least to the problem.

While the immediate impacts of climate change include displacement and infectious disease, its longer term consequences, such as air pollution and disruptions to healthcare, significantly elevate cancer risks. This makes climate change not only an environmental crisis, but also a growing oncological concern.

How do these environmental changes affect cancer patients? Cancer patients are particularly vulnerable to extreme heat and severe weather events such as hurricanes and cyclones, which disrupt healthcare access. Changes in vector ecology may lead to pandemics and infectious diseases, while food and water contamination resulting from droughts, floods and sea-level rise can cause malnutrition and gastrointestinal diseases [56].

Air pollution, particularly fine particulate matter (PM2.5), is classified as a Group 1 carcinogen by the International Agency for Research on Cancer. PM2.5 particles penetrate the lung’s terminal bronchioles, causing localised inflammation (leading to asthma and lung cancer) and can translocate into the bloodstream, triggering systemic inflammation linked to cardiovascular and neurological diseases. Notably, PM2.5-induced lung cancer affects non-smokers and emerging evidence links it to breast and other cancers [58]. Air pollution from wildfires, which are increasing in intensity and frequency due to climate change, also contains PM2.5 that is more toxic than ambient pollution [59].

Modern healthcare relies heavily on plastics, considered the signature material of the contemporary era [60]. However, plastic manufacturing accounts for 8% of global oil production, as oil serves as both a feedstock and fuel. This process disproportionately affects workers in plastics manufacturing, who experience higher cancer rates, and children in nearby communities, who suffer from elevated leukemia rates [61]. Although healthcare represents 2% of global plastic consumption, this figure is rising by 6% annually. The COVID-19 pandemic and future global health crises will likely accelerate this trend. Less than 10% of plastics are recycled globally, contributing to the accumulation of over 6 gigatons of plastic waste since 1950. Half of all plastics ever produced were made since 2002. Plastics, resistant to degradation, persist in ecosystems and biological systems as macro- and microplastics, with known carcinogenic potential [62–64]. Plastics, while vital in modern medical equipment and drug delivery, present dual risks. Not only do they contribute to environmental degradation, but the leaching of plastic byproducts into water and food supplies introduces endocrine disruptors and carcinogens, posing long-term cancer risks.

Furthermore, marine plastic pollution further threatens ocean ecosystems, which are critical for food, oxygen, livelihoods and global health [65]. Reducing reliance on fossil fuels and single-use plastics within healthcare settings can significantly mitigate both environmental harm and cancer risks, highlighting the responsibility of the medical community in combating climate change at its source.

As demonstrated by regional disparities in Brazil, Nigeria and the Philippines, the disproportionate effects of climate change on vulnerable communities underline the need for systemic mitigation strategies across oncology disciplines. A range of tools has been developed to address the environmental harms and impact of healthcare, including in cancer care, with a focus on practical, scalable solutions for reducing carbon emissions in clinical and operational settings. These tools provide evidence-based approaches that enable healthcare teams to integrate sustainability into oncology practices effectively [66, 67].

These resources have focused on the principles of reduce, reuse, recycle, rethink and research. Table 1 highlights a selection of resources in this area, including curated web-based portals that offer guidance on implementing sustainable practices across healthcare institutions.

### Surgical oncology

Surgical oncology procedures are central to the treatment of cancer but have undeniable and growing impacts on environmental sustainability. In the US, the healthcare sector accounts for 8.5% of all greenhouse gas emissions, with up to one-third generated in the perioperative environment [68]. From the production and disposal of surgical equipment, to the energy and resources required for routine operating room functions, the environmental footprint of cancer surgery spans multiple sectors. Additionally, biomedical waste from the disposal of tissues, organs and other biological materials, as well as the use of anesthetic gases (e.g., nitrous oxide and desflurane), contributes significantly to greenhouse gas emissions, with a far greater global warming potential than carbon dioxide.

These wide-ranging effects present an opportunity to address climate change within the perioperative space. A scoping review highlights various initiatives that surgeons and surgical staff can implement, including reducing perioperative greenhouse gas emissions, enhancing waste management, integrating sustainability training and education, and expanding recycling programs [69]. Operating room committees have successfully developed and executed sustainability procedures, demonstrating the feasibility of reducing perioperative waste within a short timeframe.

Further research is necessary to fully understand the environmental impact of these initiatives and to establish metrics that enable ongoing assessment of their effects on patient outcomes, cost and sustainability. There is significant potential to leverage motivated stakeholders and explore opportunities to reuse drugs, supplies and equipment, facilitating long-term reductions in the environmental footprint of surgical procedures without compromising the quality of care for patients and providers.

### Radiation oncology

Several tools have been developed to address the environmental harms and impact of healthcare, including in radiation oncology, with a focus on practical, scalable solutions for reducing carbon emissions in clinical and operational settings. One such tool is the Network Greener Calculator, introduced in recent research published in the *Journal of Clinical Oncology* [70]. This calculator was developed by analysing data from nearly 140,000 international attendees at the American Society of Clinical Oncology (ASCO) Annual Meetings, enabling conference organisers and participants to estimate emissions associated with travel and in-person attendance. The tool compares these emissions with those of virtual or hybrid conference formats. Beyond reducing carbon footprints, the calculator also addresses diversity and inclusivity by promoting equitable access to professional development opportunities that virtual and hybrid formats can provide, particularly for underrepresented or geographically remote participants [70, 71].

Another resource is the NorCal Procedural (Brachytherapy) Waste Audit Toolkit, created by Stanford Radiation Oncology resident Dr. Claire Baniel [72]. This open-access toolkit, hosted on a shared Google Drive platform, allows oncology departments to quantify, track and reduce waste associated with cancer procedures, including brachytherapy. By conducting detailed waste audits, healthcare providers can gather data on material usage and waste generation, which can then inform policy changes and improve clinical practices [73]. Early findings from these audits demonstrate practical benefits, including cost savings and reduced environmental impact, supporting more sustainable procedural oncology practices.

Building on these advancements, an External Beam Radiation Therapy Environmental Impact Calculator is under development, inspired by research published in *The Lancet Oncology* [74]. This predictive nomogram maps the carbon footprint of various treatment pathways, such as hypofractionation, and identifies opportunities to reduce emissions [75]. The tool serves dual purposes: providing clinicians with actionable data to inform sustainable decision-making and educating patients about the environmental impact of different care options. By enabling informed discussions between patients and providers, especially in cases of treatment equipoise, the tool fosters a patient-centered approach to sustainable cancer care.

Together, these tools represent significant advancements in decarbonising cancer care. They equip oncology teams with the resources needed to align high-quality care delivery with environmental stewardship, thus advancing both patient outcomes and global sustainability goals.

### Clinical trials

While radiotherapy contributes to healthcare emissions, clinical trials represent another significant area where sustainable practices can yield substantial environmental benefits. Clinical trials are essential to the development of new cancer treatments and the advancement of medical knowledge. However, clinical trials also contribute to healthcare greenhouse gas emissions. Adshead *et al* [76] estimated the contribution of clinical trials to be approximately 27.5 million tones CO_2_e, comparable to the greenhouse gas emissions of a country the size of Bangladesh.

Tools are becoming available to quantify the impact of trials, including guidance on carbon foot printing of clinical trials [77], and data highlighting common hotspots in publicly funded [78] and industry trials [79, 80]. Work is also underway to develop digital toolkits for the calculation and sharing of carbon footprinting data in both the private and public sector, which are intended to make the calculations easier and help encourage uptake. Although much work remains to make the consideration, quantification and reduction of the carbon footprint of clinical trials routine practice, initiatives such as the Concordat for the Environmental Sustainability of Research and Innovation Practice [81] in the public sector and the Sustainable Markets Initiative [82] for the pharmaceutical industry are fostering change.

Collaboration across public sector trialists, research funders, the pharmaceutical industry, healthcare systems, regulatory bodies, patients and the public in the UK and internationally will be critical to driving consistent and coordinated change. While this complex program evolves, it is essential to raise awareness of the resources and guidance already available to reduce the environmental impact of clinical trials. These include the NIHR’s Carbon Reduction Guidelines [83], the UK CRC Greener Monitoring Guidance [84] and the MRC-NIHR TMRP Greener Trials Group [85].

### Medical oncology

For medical oncology, the most significant sustainability touch point is the prescription of systemic anticancer therapy (SACT) [86]. In an assessment of 300 medical oncology protocols from Ireland’s National Cancer Control Program, over 1,200 sustainability touchpoints were identified. These included the use of dose banding, vial sharing and telemedicine to reduce travel. For instance, the carbon footprint of administering one gram of paracetamol orally is 68-fold lower than administering the same dose intravenously in glass packaging [87]. These estimates exclude additional equipment, such as giving sets and associated complications like phlebitis [88], increased plastic waste [89] and the environmental impact of pharmaceutical ingredients [90].

In medicine, low-value, unnecessary care accounts for 6%–8% of healthcare spending [91–93]. A 2017 survey of US physicians reported that 20% of overall medical care was unnecessary, including 25% of tests [94]. Movements like Choosing Wisely and Common Sense Oncology aim to reduce overtreatment, emphasising patient engagement to drive de-prescribing and de-escalation strategies [95, 96]. Overtreatment often results from evolving care standards, reimbursement systems that reward higher volumes, lack of multidisciplinary care and litigation concerns [97, 98]. In patients with metastatic disease, overtreatment at the end of life correlates with poorer quality of death [99], in-hospital mortality and higher healthcare costs [99, 100]. As a result, 30-day mortality post-SACT is now used as a key performance indicator [101]. Early referrals to palliative care are associated with less aggressive end-of-life care [102, 103], improved overall survival and quality of life [104], reduced financial burdens and lower climate impact [105, 106].

Both ASCO and the European Society of Medical Oncology (ESMO) have developed validated frameworks – the ASCO value framework and the ESMO Magnitude of clinical benefit scale – to evaluate the efficacy, toxicity and quality of life implications of anticancer therapies [107, 108]. Current prescribing practices are often linear, leading to significant drug wastage. In the US, drug wastage accounts for 4%–18% of all cancer drug spending, with mitigation strategies reducing spending by up to 17% [109–111]. For oral SACT, 25%–41% of patients report drug wastage [112–115], and among those who discontinue treatment unexpectedly, 46%–85% are left with unused doses [116]. In a Dutch study, unopened packages had a median value of €2,600 per patient [116]. Stewardship programs, modelled after antimicrobial stewardship, could be instrumental in addressing this issue [117–119].

Attributable risk factors contribute to 40% of cancer diagnoses [120]. Prioritising health promotion, screening and prevention yields both health and climate benefits. Dietary modification, for example, plays a significant role. Food systems contribute a third of global anthropogenic greenhouse gas emissions [121]. High meat consumption negatively impacts both planetary and human health through greenhouse gas emissions, water pollution and the carcinogenicity of processed meats [122]. Shifting to plant-based diets offers benefits for both health and the environment [123].

## Conclusion

**Figure 6.**
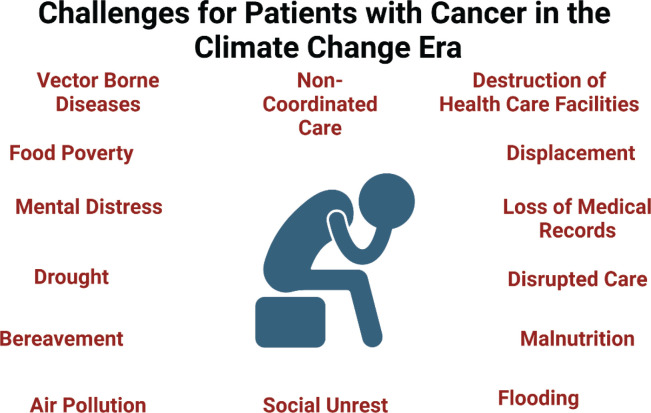
Challenges for patients with cancer in the climate change era.

The intersection of climate change and oncology presents a dual challenge: sustaining equitable, high-quality cancer care while mitigating environmental harm. As demonstrated by global disparities and regional case studies, vulnerable populations bear the heaviest burden. The aim of the current paper was to highlight the existing significant suffering experienced globally by patients (Figure 6) and the disparate challenges faced by those caring for them, and the tools available for healthcare workers in reducing the environmental impact of how we practice.

While the environmental tragedies outlined can serve as trigger events in which perception of climate risk and the need to integrate sustainability measures is more favourably viewed, they are not sufficient by themselves [124]. It has been long understood by both behavioural scientists and healthcare professionals that even highly motivated people often do not engage with actions that are consistent with their motivations. Removing or reducing the barriers that make the recommended action difficult facilitates engagement [14]. Studies of clinical trialists have demonstrated that finance, training and resourcing were identified as barriers and facilitators to climate engagement [24]. Similarly, a study of climate-engaged physicians in Canada highlighted the role of education and translational supports to guide climate impact reduction strategies in healthcare [125]. At Cancer Trials Ireland, a climate change charter has been developed by staff and patients to provide a toolkit for sustainability integration, including additional organisational touchpoints such as pension providers [67].

The resources listed in Table 1 provide a comprehensive review of some additional available toolkits, educational and literature resources. In the lifetime of the writers of this paper, cancer care has been transformed. Motivated by patient suffering, this transformation has occurred through developing multidisciplinary care pathways, through research and care pathway development. We have benefited from learning the languages of patient participation and involvement, of translational medicine, of digital technologies, among others. In an analogous manner, our community needs to engage in both climate change activism and climate change mitigation to both prevent cancer development, increase resilience in our system and a goal of moving all cancer care enterprises to net zero. Contemporaneous medical [126–129] and scientific [130–135] studies all highlight the profound impact that our triple planetary crisis has and will have, on how we live and how we practice. In March 2025, the World Meteorological Association noted that 2024 was the first calendar year to be more than 1.5^0^C above the preindustrial era, with some of the consequences becoming irreversible over hundreds if not thousands of years [136]. Learning the language of planetary health and integrating it into how we care for patients is central to addressing this crisis if we are to continue to maintain and improve cancer outcomes in all of our communities.

## Conflicts of interest

No conflicts of interest to declare.

## Funding

No funding received for this study.

## Author contributions

Conceptualisation: SOR, JS, ESH, EC.

Data Curation: n/a.

Formal Analysis: n/a.

Investigation: n/a.

Methodology: all authors.

Project Administration: CF, CH, KR, EC, SOR.

Resources: CH, KR.

Software: CH, KR.

Supervision: SOR, CH, KR.

Validation: all authors.

Visualisation: all authors.

Writing-Original: CF, RDG, OA, JHS, ESH, LF, GG, SOR.

Writing – review and editing: all authors.

Graphics: SOR under licence to biorender.

## Figures and Tables

**Table 1.** Resource toolkits.

**Surgical oncology** 1. Gorgun, E et al. **Environmental sustainability initiatives in the operating room: a scoping review** Ann Surg Open 5(3):e451 [69]. *A comprehensive review of sustainability initiatives in operating rooms, including waste reduction, energy efficiency, and environmentally friendly procurement.* 2. Ewbank, C et al. **The development of a surgical care and climate change matrix: a tool to assist with prioritization and implementation strategies**. Ann Surg. 2021;273(2):e50–e51 [137]. *Introduces a matrix to help surgical teams integrate climate-conscious practices into surgical care.* 3. **Royal college of surgeons of England – Intercollegiate Green Theatre Checklist** [138]. *A structured checklist for reducing waste and energy consumption in surgical theatres.* 4. **Centre for sustainable healthcare – surgical care sustainability network** [139]. *A UK-based network supporting hospitals and surgical teams in implementing sustainability in surgical care.*
**Radiation oncology** 1. Lichter, KE et al. **Reducing the environmental impact of health care conferences: a study of emissions and practical solutions**. JCO Glob Oncol. 2024;10:e2300209 [70]. *Highlights the carbon footprint of healthcare conferences and offers strategies for reducing emissions.* 2. Baniel, CC. **NorCal procedural (Brachytherapy) waste audit toolkit** (Resource) [72]. *A practical, open-access tool for evaluating and reducing procedural waste in brachytherapy.* 3. Lichter, KE et al. **Quantification of the environmental impact of radiotherapy and associated secondary human health effects: a multi-institutional retrospective analysis and simulation**. Lancet Oncol. 2024;25(6):790–801 [74]. *Evaluates the carbon footprint of radiotherapy and models its broader environmental health impact.* 4. Dupraz, C et al. **The carbon footprint of external beam radiotherapy and its impact in health technology assessment**. Clin Transl Radiat Oncol. 2024; 48:100834 [140]. *Provides insights into carbon emissions from external beam radiotherapy and mitigation strategies.* 5. Piffoux, M et al. **Insights on the carbon footprint of radiotherapy in France**. Cancer Radiother. 2023;27(6-7):487–90 [141]. *Examines the environmental impact of radiation therapy in the French healthcare system.* 6. Ali, D; Piffoux, M. **Methodological guide for assessing the carbon footprint of external beam radiotherapy: a single-center study with quantified mitigation strategies**. Clin Transl Radiat Oncol. 2024;46:100768 [142]. *A guide for oncology centers to measure and reduce their radiotherapy-related emissions.*
**Clinical trials** 1. National Institute for Health and Care Research. **NIHR carbon reduction guidelines 2019** [83]. *UK guidelines for reducing the carbon footprint of clinical trials.* 2. UKCRC Registered Clinical Trials Units. **Recommendations for undertaking greener monitoring** [84]. *Guidance on reducing emissions related to trial monitoring and management.* 3. TMRP Greener Trials Group. **Enabling lower carbon clinical trials** [85]. *Resources for making clinical trials more environmentally sustainable.*
**Medical oncology** 1. Nagarajah, S et al. **Implementation and impact of choosing wisely recommendations in oncology**. JCO Oncol Pract 2022. 18, 703–712 [95]. *Examines the effectiveness of de-prescribing and reducing unnecessary treatments.* 2. Booth, CM et al. **Common sense oncology: outcomes that matter.** Lancet Oncol. 2023;24(8):833–5 [96]. *Advocates for value-driven cancer care and sustainability.* 3. Cherny, NI et al. **ESMO-Magnitude of clinical benefit scale version 1.1.** Ann Oncol. 2017;28(10):2340–66 [107]. *This tool helps assess the clinical benefit of oncology drugs to prioritize treatments that provide meaningful patient outcomes.* 4. ASCO. **Value framework net health benefit worksheet: advanced disease setting** [108]. *A decision-support tool to help balance clinical benefit with resource utilization.* 5. Bernicker E et al. **Climate change and cancer care: a policy statement from ASCO**. JCO Oncol Pract 2024. 20, 178–186 [10]. *ASCO’s official stance on integrating sustainability into oncology practice.* 6. Lynch E et al. **Why we should, and how we can, reduce the climate toxicity of cancer care**. JCO Oncol Pract 2024. OP2400680 [86]. *Discusses the environmental impact of cancer care and outlines practical strategies to reduce the carbon footprint of oncology practices.* 7. ESMO Climate Change Task Force** – Resources from ESMO 2023 and 2024 sessions including slide sets. #ESMO4Climate Portal of resources for healthcare professionals** [143]. *Provides educational resources, expert discussions, and actionable strategies for integrating sustainability into oncology care, with a focus on mitigating the impact of climate change on cancer treatment and patient outcomes.*
**Institutional and policy resources** 1. Carlson, RW et al. **NCCN framework for resource stratification: a framework for providing and improving global quality oncology care. J Natl Compr** Canc Netw. 2016; 14(8):961–9 [51]. *A tiered resource-stratified framework for oncology care, useful in disaster response and low-resource settings*. 2. **National Institutes of Health Climate Change and Human Health Literature Portal** [144]. *A curated database of global research on climate change and health, supporting evidence-based policy and practice.* 3. **Healthcare without harm europe** [145]. *A network promoting sustainability in healthcare through projects, scalable solutions, and collaboration.* 4. **Healthcare without harm climate impact checkup online course** [146]. *An online course guiding healthcare professionals in emissions tracking and carbon management planning.* 5. **Joint Commission International** [147]. *A global accreditation body assisting hospitals in integrating sustainability while maintaining quality and patient safety.* 6. **Geneva sustainability centre toolbox** [148]. *A resource hub providing guidelines and case studies to help hospitals develop climate-resilient healthcare systems.* 7. **Global green and healthy hospitals** [149]. *A global initiative helping hospitals implement environmentally sustainable practices.*
**Professional and clinical guidelines** 1. Irish college of general practitioners – Glas Toolkit [150]. *Practical sustainability recommendations for general practitioners.* 2. **Royal college of physicians London – green physician toolkit** [151]. *A toolkit focusing on reducing environmental impact in clinical practice.* 3. **Royal college of general practitioners – green impact for health toolkit** [152]. *A structured guide for improving sustainability in primary care in the UK.* 4. **Canadian association of physicians for the environment – climate change toolkit for health professionals** [153]. *Education on climate change and health, with actionable mitigation strategies.* 5. **Irish doctors for the environment – a guide to being a green prescriber: Inhalers** [154]. *A guide on the environmental impact of inhalers and strategies for optimizing their use in clinical practice.* 6. **Sustainability in radiology: position paper and call to action** [155]. *A multi-society position paper outlining strategies to promote environmental sustainability across global radiology practices.* 7. **Reducing emissions from conferences and meetings** [156]. *A behavioral strategy paper offering guidance on reducing greenhouse gas emissions from scientific conferences and professional meetings.* 8. **Cross- and interdisciplinary climate research framework** [157]. *A framework describing how research centers can promote integrated, interdisciplinary approaches to climate science and collaboration.*
